# High Proportions of Patients With Advanced HIV Are Antiretroviral Therapy Experienced: Hospitalization Outcomes From 2 Sub-Saharan African Sites

**DOI:** 10.1093/cid/ciy103

**Published:** 2018-03-04

**Authors:** Janet Ousley, Aline Aurore Niyibizi, Stephen Wanjala, Alexandra Vandenbulcke, Beatrice Kirubi, Willis Omwoyo, Janthimala Price, Leon Salumu, Elisabeth Szumilin, Sofie Spiers, Gilles van Cutsem, Maria Mashako, Freddy Mangana, Ramzia Moudarichirou, Rebecca Harrison, Tony Kalwangila, Gisele Lumowo, Vincent Lambert, David Maman

**Affiliations:** 1 Médecins Sans Frontières, Paris, France; 2 Médecins Sans Frontières, Brussels, Belgium; 3 Epicentre, Paris, France

**Keywords:** Kenya, Democratic Republic of Congo, treatment failure

## Abstract

**Background:**

Human immunodeficiency virus (HIV) remains an important cause of hospitalization and death in low- and middle- income countries. Yet morbidity and in-hospital mortality patterns remain poorly characterized, with prior antiretroviral therapy (ART) exposure and treatment failure status largely unknown.

**Methods:**

We studied HIV-infected inpatients aged ≥13 years from cohorts in Kenya and the Democratic Republic of Congo (DRC), assessing clinical and demographic characteristics and hospitalization outcomes. Kenyan inpatients were prospectively enrolled during hospitalization; identical retrospective data were extracted for Congolese patients meeting the study criteria using routine medical information.

**Results:**

Among 338 HIV-infected patients in Kenya and 411 in DRC, 83.7% (95% confidence interval [CI], 79.4%–87.3%) and 97.3% (95% CI, 95.2%–98.5%), were admitted with advanced disease (defined as CD4 <200 cells/µL or World Health Organization stage 3/4 illness). Among inpatients with advanced HIV, 35.4% and 21.7% were ART-naive at admission. Patients under care had a median time of 44.1 (interquartile range [IQR], 18.4–90.5) months and 55.9 (IQR, 28.1–99.6) months on treatment; 17.2% (95% CI, 13.5%–21.6%) and 29.6% (95% CI, 25.4%–34.3%) died, 25.9% (95% CI, 16.0%–39.0%) and 22.5% (95% CI, 15.8%–31.0%) of these within 48 hours.

**Conclusions:**

Across 2 diverse clinical contexts in sub-Saharan Africa, advanced HIV inpatients were frequently admitted with low CD4 counts, often failing first-line ART. Earlier identification of treatment failure and rapid switching to second-line ART are needed.

Human immunodeficiency virus (HIV) persists as a major cause of hospitalization and in-hospital mortality in low- and middle-income countries (LMICs), where much of the world’s HIV prevalence occurs [1, 2]. Despite having increased access to antiretroviral therapy (ART) for more than a decade in these countries, many HIV-infected inpatients continue to present late to care with advanced disease (defined as a CD4 count <200 cells/µL or World Health Organization [WHO] stage 3/4 illness) after having never sought testing or initiated ART [3, 4]. Others are hospitalized after years on ART and are admitted in a state of clinical, immunologic, and virologic failure, with disease that is resistant to ART due to myriad factors that often include drug adherence challenges, which prevent consistent access to and use of HIV therapies. Indeed, studies have shown that up to 25% of patients will interrupt their treatment at some point [5].

Morbidity and mortality among hospitalized patients with advanced HIV remain poorly understood in settings with few laboratory or diagnostic resources, with little evidence characterizing these patients’ prior ART exposure and treatment failure status [6]. To better understand these patients, we performed separate hospital-based analyses of HIV-infected individuals admitted to inpatient departments in facilities supported by Médecins Sans Frontières (MSF) in 2 different settings in Kenya and the Democratic Republic of Congo (DRC).

## METHODS

### Study Setting

This operational cohort study was conducted at 2 live clinical sites using medical data from inpatient departments in MSF-supported hospital wards. In Kenya, Homa-Bay County (HBC) has the highest adult HIV prevalence in the country (27.1%) and one of the highest rates in the world [7]. As the first place in Kenya to introduce ART in a public medical facility, HBC also achieved an estimated 63% ART coverage in 2015 [8]. MSF has supported the inpatient department in HBC hospital since 2014. Kinshasa, the capital of DRC, has a comparatively low HIV prevalence (1.2%), yet <20% of the city’s health centers offer HIV services (including prevention, testing, and some free care), and only 33% (95% confidence interval [CI], 26%–40%) of the HIV-infected population in DRC is estimated to be on ART [9]. In Kinshasa, MSF has been providing HIV and tuberculosis (TB) care at the Centre Hospitalier de Kabinda since 2002, offering free ambulatory services, treatment, and inpatient care. The inclusion of 2 cohorts for analysis was not intended to be comparative but rather to report inpatient trends across 2 different sub-Saharan clinical environments where little or no prior evidence has been published about advanced HIV patients.

### Study Design and Data Collection

Using a mixed methods design, we collected data prospectively at the HBC site while retrospectively extracting identical data elements from the Kinshasa cohort, representing 2 distinct African HIV contexts: a periurban, middle-income, stable setting with relatively high ART coverage in Kenya, and an urban setting with low ART coverage in a low-income, fragile health system in the DRC. In Kenya, study participants were recruited and provided consent upon admission to inpatient care in a larger public hospital facility hosting those with HIV and other medical conditions. In DRC, the specialty hospital admitted only the HIV-infected, and de-identified data from all admitted patients meeting study criteria were selected and extracted from clinical databases. In both sites, participants were ≥13-year-olds admitted to care with HIV-1 disease from January to March 2015 (Kenya) and May to July 2017 (DRC). To establish postdischarge mortality rates, a subset of 168 Kenyan patients received a follow-up phone interview 9 months after being released from hospital care. Due to contextual limitations, the corresponding data were unavailable in Kinshasa.

### Clinical Procedures and Laboratory Methods

In both countries, a hemogram was performed upon admission for each study participant. Sputum specimens were examined for *Mycobacterium tuberculosis* using fluorescent microscopy or GeneExpert RIF/MTB (Cepheid, Sunnyvale, California) or urine lipoarabinomannan assay (TB-LAM) for rapid testing. Malaria antigens were investigated using the SD Bioline Rapid Diagnostic Test, and cryptococcal antigen (CrAg) lateral flow assay was used to detect *Cryptococcus* in blood and cerebrospinal fluid. CD4 cell counts were determined by Alere Pima PoC test (Alere Health sprl, St Denis, Belgium) in Kenya and by FACSCount (BD Biosciences, San Jose, California) in Kinshasa. For patients with suspected treatment failure, HIV-1 RNA was quantified as part of routine care in Kinshasa with Abbot m2000 RealTime HIV-1 (Abbot Molecular). In Kenya, this was done for a subset of patients through a private laboratory (Lancet Laboratories, Kenya PLK) due to operational and contextual constraints.

### Statistical Analysis

In Kenya, data were stored in an electronic data capture tool customized for the study (Redcap, Harvard University, Boston, Massachusetts), while in DRC it was extracted from the MSF patient database into EpiInfo. All analysis was conducted using Stata version 14. Baseline sociodemographic and HIV treatment characteristics (ART, CD4, viral load [VL]) were assessed using the mean with standard deviation or the median with interquartile range (IQR) for continuous variables, and frequencies and proportions for categorical data. Tests for differences in characteristics by sex and time on ART were performed using *t* tests or χ^2^ analysis. Univariate and multivariate regression models assessed risk factors for mortality, including CD4 levels, opportunistic infections, and viremia. Clinical and immunological failures were described using WHO definitions. Aggregate measures were not calculated and comparative analysis between the 2 sites was not conducted.

### Ethics Committee Approval

In Kenya, ethics approval was granted from the Kenya Medical Research Institute national research committee (protocol number 450). Each participant (or their guardian if incapacitated) provided written consent to participate to the study. In DRC, the Kinshasa Ethical Review Board (ERB) of the Ministry of Health provided approval of study protocols and procedures, but verbal consent from patients was not sought for the retrospective analysis of routinely collected, anonymized data. MSF’s organizational Ethics Review Board provided additional review of both sites’ study procedures.

## RESULTS

### Baseline Clinical Characteristics

Demographic and clinical characteristics of 338 HIV-infected patients in Kenya and 411 HIV patients in DRC (median CD4 at presentation, 122 [IQR, 44.5–323] cells/µL and 68.5 cells/µL [IQR, 23–215], respectively) are shown in Table 1. Of these, 83.7% and 97.3% were admitted with advanced disease, among whom 45.3% and 54.3%, respectively, were admitted with a CD4 count of <100 cells/μL. More Kenyan men than women presented with advanced disease (80.0% vs 68.0%, *P* = .02) with substantially lower median CD4 counts (93 [IQR, 42–234] cells/μL vs 172 [IQR, 46–409] cells/μL for women). In DRC, no gender differences were seen. Of all HIV-infected inpatients, 53.2% and 43.6% were “late presenters,” defined as individuals who had either never initiated ART (including those with and without a prior HIV diagnosis) or who had only recently initiated treatment (<6 months on ART). Late presenters are further characterized in Tables 1–3.

**Table 1.** Comparison of Sociodemographic and Clinical Characteristics Between Antiretroviral Therapy (ART)–Naive and ART-Experienced Human Immunodeficiency Virus–Infected Inpatients at Admission

	Kenya (n = 331)^a^			DRC (n = 376)^a^		
	Late Presenters^b^		ART >6 mo	Late Presenters^b^		ART >6 mo
Characteristic	No Prior ART	ART <6 mo		No Prior ART	ART <6 mo	
Age, y						
13–29	51 (43.6)	20 (33.9)	42 (27.1)	17 (20.7)	4 (4.9)	36 (17.1)
30–49	55 (47.0)	33 (55.9)	85 (54.8)	52 (63.4)	56 (68.3)	127 (60.2)
≥50	11 (9.4)	6 (10.1)	28 (18.1)	13 (15.9)	22 (26.8)	48 (22.8)
Sex						
Male	50 (42.7)	33 (55.9)	78 (50.3)	27 (33.3)	29 (35.4)	67 (31.6)
Female	67 (53.3)	26 (44.1)	77 (49.7)	54 (66.7)	53 (64.6)	145 (68.4)
WHO stage						
1/2	46 (39.2)	10 (17.0)	30 (19.5)	3 (3.8)	2 (2.5)	6 (3.0)
3/4	71 (60.6)	124 (80.0)	124 (80.5)	76 (96.2)	79 (97.5)	193 (97.0)
CD4 count, cells/µL						
>500	25 (21.4)	4 (6.8)	19 (12.3)	4 (5.2)	3 (4.0)	10 (5.2)
200–499	22 (18.7)	14 (23.7)	35 (22.5)	12 (15.6)	17 (22.7)	47 (24.6)
100–199	18 (15.3)	11 (18.6)	29 (18.7)	5 (6.5)	12 (16.0)	32 (16.8)
<100	51 (43.6)	30 (50.8)	71 (45.8)	56 (72.7)	43 (57.3)	102 (53.4)
HIV-1 RNA quantification						
Result available, No.	…	…	54	52	60	171
VL >1000 copies/mL	…	…	63.0%	47 (90.4)	22 (36.7)	106 (62.0)
Knowledge of HIV status						
Undiagnosed at admission	47 (40.1)	…	…	NA^d^	…	…
Treatment interruptions^c^	…	0	7	…	6	40
ART regimen						
NNRTI-based (first line)	…	59	140 (90.3)	…	80 (100)	156 (75.4)
PI-based (second line)	…	0	15 (9.7)	…	0 (0)	51 (24.6)
Total	117	59	155	82	82	212

Data are presented as No. (%) unless otherwise indicated.

Abbreviations: ART, antiretroviral therapy; DRC, Democratic Republic of Congo; HIV, human immunodeficiency virus; NA, not applicable; NNRTI, nonnucleoside reverse transcriptase inhibitor; PI, protease inhibitor; VL, viral load; WHO, World Health Organization.

^a^Seven patients in Kenya and 35 in DRC not included in totals because of missing ART start date.

^b^ART-naive late presenters include both those who had and had not been previously diagnosed.

^c^Self-reported for any reason including drug toxicity, sociocultural reasons (fear, stigma, etc), stockouts, adherence.

^d^Not applicable; all patients are diagnosed at admission as it is an HIV hospital only.

**Table 2.** Primary Comorbidities at Admission and Case Fatality Rates Among Antiretroviral Therapy (ART)–Naive and ART-Experienced Human Immunodeficiency Virus–Infected Inpatients

Comorbidity	Kenya (n = 338)						DRC (n = 376)					
	Late Presenters^a^				ART Experienced		Late Presenters^a^				ART Experienced	
	ART-Naive	CFR	ART <6 mo	CFR	ART >6 mo	CFR	ART-Naive	CFR	ART <6 mo	CFR	ART >6 mo	CFR
Tuberculosis	20 (17.1)	25.0	21 (33.9)	28.6	38 (24.5)	26.3	55 (67.1)	30.9	63 (76.8)	45.9	134 (63.2)	25.0
CCM	6 (5.1)	50.0	8 (13.6)	25.0	10 (6.5)	20.0	8 (9.8)	12.5	8 (9.8)	37.5	21 (9.9)	9.5
PCP	0	NA	0	NA	0	NA	5 (6.1)	60.0	7 (8.5)	50.0	13 (6.1)	46.2
Malaria	25 (21.4)	0	6 (10.2)	33.3	23 (14.8)	4.4	12 (14.6)	27.3	14 (28.6)	28.6	34 (16.0)	23.5
Toxoplasmosis	2 (1.7)	0	0	NA	1 (0.6)	0	19 (23.2)	36.8	19 (23.2)	38.9	28 (13.2)	46.4
Kaposi sarcoma	0	…	2 (1.7)	0	6 (3.9)	33.3	2 (2.4)	50	2 (2.4)	50	4 (1.9)	75.0
Gastroenteritis	21 (17.9)	19.1	10 (16.9)	10.0	25 (16.1)	4.0	…	…	…	…	…	…
Bacterial meningitis	11 (9.4)	27.3	6 (10.2)	83.3	12 (9.4)	25.0	…	…	…	…	…	…

Data are presented as No. (%) unless otherwise indicated. Case fatality rate is defined as fatalities occurring while hospitalized.

Abbreviations: ART, antiretroviral therapy; CCM, cryptococcal meningitis; CFR, case fatality rate; NA, not applicable; PCP, pneumocystis pneumonia.

^a^ART-naive late presenters include both those who had and had not been previously diagnosed.

**Table 3.** Comparison of Viremia Among Patients, by Treatment Duration and Clinical/Immunological Characteristics

	Detectable VL (> 1000 Copies/mL)		
Characteristic	ART Naive (Late Presenters^a^)	ART <6 mo (Late Presenters^a^)	ART >6 mo (ART Experienced)
Kenya^b^			
WHO stage 3 or 4^c^	NA	NA	33 (64.7)
CD4 count <100 cells/μL	NA	NA	31 (83.8)^d^
DRC			
WHO stage 3 or 4^c^	46 (90.2)	22 (37.3)	105 (63.5)
CD4 count <100 cells/μL	35 (92.1)	13 (43.3)	67 (80.7)

Data are presented as No. (%) unless otherwise indicated.

Abbreviations: ART, antiretroviral therapy; DRC, Democratic Republic of Congo; NA, not applicable; VL, viral load; WHO, World Health Organization.

^a^ART-naive late presenters include both those who had and had not been previously diagnosed.

^b^Viral load done only during the last month of the study and only on patients on ART >6 months.

^c^Defined as WHO stage 3/4 illness at admission to inpatient care.

^**d**^Immunological failures.

Among Kenyan and Congolese patients, 35.3% and 21.8%, respectively, were ART naive at admission. Among those on ART >6 months at admission (46.8% and 56.4%), patients had a median time of 44.1 months [IQR, 18.4–90.5] and 55.9 [IQR, 28.1–99.6] months on treatment in Kenya and DRC, respectively. Overall, patients were hospitalized for a median of 6 days in both sites. Median CD4 counts were no different in Kenya (114 [IQR, 36–309] cells/μL in ART experienced vs 129 [IQR, 46–337] cells/μL in ART naive) and higher in DRC among the ART-experienced patients (84 [IQR, 22–239] cells/μL vs 47 [IQR, 22–123] cells/μL). Of Kenyan patients, 26.9% ever had tuberculosis and 9.8% were on TB treatment at admission. In DRC, 27.7% ever had tuberculosis and 28.2% were on TB treatment at admission.

### Nonnucleoside Reverse Transcriptase Inhibitor First-line Treatment Failure

Among patients on ART for >6 months, 93.2% and 74.3% were on nonnucleoside reverse transcriptase inhibitor–based regimens in Kenya and DRC, respectively. Patients admitted to care in a state of clinical (WHO stage 3/4), immunological (CD4 count <100 cells/μL plus ART >6 months), or virological failure (VL >1000 copies/mL) are detailed in Table 1.

### Morbidity

Primary coinfections and case fatality rates for each morbidity are characterized in Table 2. Comorbidity was associated with lower CD4 levels at admission, especially among those with TB. Infectious illness caused most hospitalization: TB (13.3%; median hospitalization of 6 days), malaria (11.6%; 3.5 days), pneumonia (8.9%; 5 days), gastroenteritis (9.9%; 6 days), and neurological infections (toxoplasmosis, cryptococcal meningitis [CCM], and meningitis) were principal infections in Kenya. In Kinshasa, TB (67.2%; 7 days), malaria (16.1%; 5 days), toxoplasmosis (17.1%; 7.5 days), CCM (9.4%; 9.5 days), and pneumocystis pneumonia (8.7%; 10 days) were most frequent.

Among Kenyan and Congolese HIV patients admitted with TB, median CD4 count at admission was 79 (IQR, 46–227) cells/μL and 63 (IQR, 19–171) cells/μL, respectively. The majority of TB cases were pulmonary in Kenya (70.0%) but extrapulmonary in DRC (80.8%), with most extrapulmonary TB cases manifesting as meningitis (24.0%) and pleural effusions (16.0%) in Kenya and as disseminated (82.5%) and meningitis (14.3%) in DRC. Among CCM patients (n = 20 and n = 39, respectively), all were diagnosed with a median CD4 count of <200 (38 [IQR, 25.5–68.5] cells/μL in Kenya and 43 [IQR, 4–74.5] cells/μL in DRC) and 83.3% and 91.6% of these had a CD4 count of <100 cells/μL.

### Mortality

**Figure 1.**
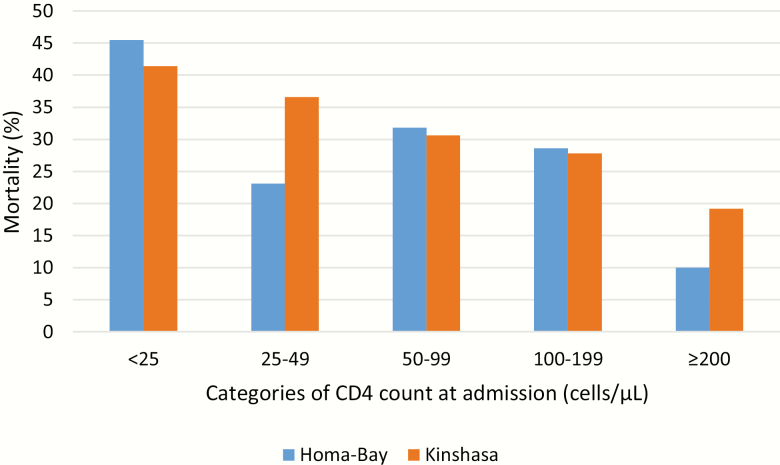
Mortality among patients diagnosed with tuberculosis, stratified by CD4 cell count at admission, Homa-Bay, Kenya (n = 80) and Kinshasa, Democratic Republic of Congo (n = 248).

In both sites, CD4 cell count remained the most significant determinant of mortality for all groups, regardless of sex, age, or type or number of comorbidities (Table 2 and Figure 1). Overall, 17.2% of Kenyan and 29.6% of Congolese HIV-infected inpatients died while hospitalized. Within this group, 22.4% and 25.8% of deaths occurred within 48 hours of admission and 93.1% and 81.2% within 2 weeks. After a median observation time of 9 months, a further 30.4% of Kenyan patients died after being discharged from hospital. Postdischarge information was unavailable in Kinshasa. In both sites, CD4 <50 cells/μL at admission increased the odds of mortality, though men in Kenya (adjusted odds ratio, 3.0 [95% CI, 1.6–6.0]) had an elevated mortality risk that was not seen in Kinshasa.

Tuberculosis, the most common cause of hospitalization, occurred in 21.2% and 67.2% of the patients, and in 36.2% and 74.2% of deaths. Among the extremely immunosuppressed, TB was also by far the largest cause of mortality: 22.4% and 28.3% of deaths were among those admitted with <25 cells/μL, 19.0% and 15.0% among those with 25–49 cells/μL, and 13.8% and 20.0% among those with >350 cells/µL, in Kenya and DRC, respectively (Figure 1). Reduced CD4 count at admission was also associated with those whose TB caused their hospitalization. Beyond TB, neurological infections were the most important causes of death in Kenya and in DRC.

## DISCUSSION

Our findings present 2 diverse inpatient contexts in sub- Saharan Africa where, nevertheless, similar characteristics and outcomes emerged, particularly for those with advanced HIV. HIV-infected inpatients were often hospitalized extremely immunosuppressed (45.9% and 56.6% with CD4 <100 cells/µL), usually because of HIV-related infectious illnesses that could have been diagnosed prior to hospitalization. The 2 contexts were very different: an urban, private MSF hospital in a low-prevalence, low-ART coverage fragile health system vs a rural, public hospital (supported by MSF) in a high-prevalence, middle-income country with high ART coverage (especially among women). Though the 2 contexts cannot be directly compared, across the 2 settings many patients had prior exposure to ART (only one-third in Kenya and one-quarter in DRC were ART naive at admission) and were admitted in a state of clinical, immunological, or virological failure (80.5%, 45.8%, and 63.2%, respectively, in Homa-Bay and 97.0%, 53.4%, and 62.0%, respectively, in Kinshasa). Regardless of the sites’ differences, these HIV patients were exposed to health systems that failed to identify and address their worsening conditions. To our knowledge, this is the first analysis to report hospitalization outcomes for adult HIV-infected inpatients from a Kenyan or Congolese setting [10].

In 2015 alone, 1.1 million individuals still died due to AIDS, despite being in an age of free ART that is more available and effective than ever before [11]. The Kenyan and Congolese inpatients studied here were different than patients seen a decade ago in that they were aware of their HIV status (85.6% of Kenyan inpatients) and had significant exposure to ART (often years) [12–14]. This new type of advanced HIV inpatient highlights how much more must be done. Recognizing those with deteriorating conditions earlier and responding to them more quickly (including at the primary care level and in the community where obstacles related to extreme stigma, health literacy, and psychosocial issues persist) could likely prevent many advanced cases. It would also address some of the treatment adherence and retention-in-care issues that cause advanced HIV.

CD4 cell counts at admission remained the single most important predictor of morbidity, mortality, and length of stay in both contexts, confirming research that HIV-infected inpatients admitted with low CD4 die more quickly (up to 15 years shorter life expectancy), more frequently, and with more complicated coinfections [15–19]. Of Kenyan and Congolese patients on ART >6 months, 45.8% and 67.2%, respectively, were admitted in a state of immunological failure, and 80.7% and 83.8% of these patients also had a detectable VL of >1000 copies/mL, suggesting either adherence problems or disease that was already resistant to treatment. Yet, during the course of this research, few patients switched to second-line drugs while hospitalized regardless of their CD4 or VL, often because there was not enough information available about adherence and resistance in these settings. Indeed, research from LMICs overall show that patients may take as long as 21 months to switch even after virologic failure has been confirmed [20].

Direct comparison of these different cohorts should be avoided. Though the data are presented together, complete standardization was limited by data availability and quality in each operational context: Kenyan patients were in a public general hospital whereas Congolese patients were in a specialized HIV facility (though all received MSF’s standard of care), and the data come from slightly different time periods. Other possible residual confounders related to differences in prevalence, ART coverage, health system resources, socioeconomic status, health-seeking behaviors, or even seasonality (particularly in Kenya where patients were largely agrarian) in the 2 sites. VL testing was unavailable for all suspected treatment failures (in Kenya, 59.9% of patients with clinical failure did not have results), and those receiving VL testing may have been selected based on contextual factors. HIV-1 genotyping, and thus conclusions about resistance patterns, were not possible. Finally, inpatients are likely not representative of the larger population where individuals remain undiagnosed, have unequal access to care, or may die in their communities before reaching a health facility.

## CONCLUSIONS

The large majority of patients in our cohorts had a prior history of ART treatment when they were admitted to inpatient care. Some of these patients were likely experiencing treatment failure and needed their drug regimen to be quickly changed. Mortality among HIV-infected inpatients was high in both Kenya and the DRC, as many patients presented extremely sick and died often and quickly, often needing intensive care services that were beyond what was available in MSF-supported inpatient departments. MSF-supported hospitals usually have relatively high-quality care, consistent funding and resources, and patients with better access to services and ART. The fact that these results were seen even in MSF’s facilities implies that addressing advanced HIV will demand more: improved identification of treatment failure and symptomatic HIV patients in primary healthcare facilities and in the community, better point-of-care diagnostic technology (eg, TB-LAM and CrAg tests for opportunistic infections, or oral HIV self-tests), and patient tracing and psychosocial support for people who drop out of care or stop treatment [21]. The need remains for expanded access to routine VL testing to verify patients’ adherence and treatment efficacy, and for CD4 as an indicator of immunocompromise to better target opportunistic infection testing. Hospitals will need adapted clinical and counseling approaches, including more timely identification of treatment failures, adherence issues, and adapted care and prophylaxis protocols. Time is critical and reactivity essential.

Though our cohorts cannot be directly compared to each other or other studies, the trends found in each of these contexts clearly indicate that AIDS deaths still haunt the inpatient wards in these sub-Saharan contexts. Despite impressive investments and advancements against HIV over 3 decades, the fight is not over.

## Notes

***Author contributions.*** D. M., A. N., G. v. C., F. M., and S. S. conceived of the study; participated in its design, coordination, and statistical analysis; and drafted the manuscript. J. O. participated in the design and statistical analysis; contributed to the manuscript; and provided technical review of the manuscript. F. M. participated in the design and data collection of the study and technical review of the manuscript. A. N., B. R., J. P., F. M., R. M., T. K., and G. L. supported data collection, data cleaning, and preliminary analysis. D. M. additionally oversaw the study and provided final technical and editorial review of the manuscript. All other authors provided rigorous technical review.

***Financial support.*** This work was supported by MSF program funds. All authors contributed to this study as part of regular duties as staff with MSF. This supplement was supported by funds from the Bill & Melinda Gates Foundation.

***Potential conflicts of interest.*** All authors: No reported conflicts of interest. All authors have submitted the ICMJE Form for Disclosure of Potential Conflicts of Interest. Conflicts that the editors consider relevant to the content of the manuscript have been disclosed.

***Supplement sponsorship.*** This article appears as part of the supplement “Advanced HIV Disease,” sponsored by the World Health Organization.
